# Effectiveness of community-based Baduanjin exercise intervention for older adults with varying frailty status: a randomized controlled trial

**DOI:** 10.1186/s11556-024-00363-6

**Published:** 2024-10-10

**Authors:** Nien Xiang Tou, Siew Fong Goh, Susana Harding, Mary Ann Tsao, Tze Pin Ng, Shiou-Liang Wee

**Affiliations:** 1 Geriatric Education and Research Institute (GERI), 2 Yishun Central 2, Tower E Level 4 GERI Admin, Singapore, 768024 Singapore; 2 Tsao Foundation, Singapore, Singapore; 3 S R Nathan School of Human Development, Singapore University of Social Sciences, 463 Clementi Road, Singapore, 599494 Singapore

**Keywords:** Physical function, Community exercise program, Frailty, Qigong, Older adults

## Abstract

**Background:**

Due to poorer exercise tolerance, it may be challenging for frail older adults to engage in moderate- or vigorous-intensity exercise. While low-intensity exercise interventions may be more feasible, its effectiveness for such population group remains unclear. We examined the effectiveness and implementation of community-based Baduanjin Qigong, a low-intensity exercise program in older adults with varying frailty status.

**Methods:**

A two-arm, multicenter assessor-blind parallel group randomized controlled trial was conducted at three local senior activity centers. Fifty-six community-dwelling older adults with low handgrip strength were randomly allocated to either the intervention (IG) or wait-list control (CG) group. The IG underwent a supervised 16-week Baduanjin exercise program at a frequency of 2–3 × 60 min sessions/week. The CG was instructed to maintain their usual activity and received a monthly health education talk. The primary outcome measures were knee extension strength, vital exhaustion, and fear of falling. Secondary outcome measures include physiological falls risk, handgrip strength, gait speed, timed up and go test, 30-second sit-to-stand, quality of life, depression, and frailty. All outcome measures were assessed at baseline and 4-month follow-up.

**Results:**

Overall, there were no statistically significant differences in all outcome measures between CG and IG at 4-month follow-up. However, in exploratory compliance analysis, a statistically significant group x time interaction was found for vital exhaustion (B = -3.65, 95% CI [-7.13, -0.16], *p* = .047) among participants with at least 75% attendance. In post-hoc within-group comparisons, IG showed improved vital exhaustion by 4.31 points (95% CI [1.41, 7.20], *d* = 0.60). The average participant attendance rate was 81.3%. No major adverse events occurred, and all participants reported positive experiences with the exercise intervention.

**Conclusions:**

Our study demonstrated that Baduanjin is a safe, feasible, and acceptable exercise program that can be successfully implemented in community settings for older adults with varying frailty status. With good adherence, Baduanjin exercise could potentially be effective in alleviating vital exhaustion. However, the effectiveness of Baduanjin on physical performance, psychological measures and frailty in community-dwelling older adults remains equivocal.

**Trial registration:**

ClinicalTrials.gov NCT04549103. Registered September 16, 2020.

## Background

The global population is ageing rapidly, with a concomitant rise in the number of older adults experiencing age-related conditions. Frailty, characterized by a decline in physiological reserves and increased vulnerability to stressors [1], is increasingly prevalent with advancing age. The estimated weighted average prevalence of physical pre-frailty and frailty among community-dwelling older adults was 41.6% and 10.7%, respectively [2]. Given that frailty is associated with increased risk of adverse health outcomes including disability, hospitalization, institutionalization and mortality [3], it is a key geriatric syndrome that poses significant public health concern [4, 5].

Frailty is a dynamic process marked by shifts between frailty states [3, 6, 7]. A recent meta-analysis reported that a change in frailty state was observed in 42.8% of community-dwelling older adults within a mean follow-up period of 3.9 years [8]. While there is greater likelihood of deterioration over time, frailty can potentially be improved over time as well [7, 8]. This has led to experts’ consensus that frailty is reversible with appropriate interventions [9, 10].

Exercise has long been established as a cornerstone of effective interventions to manage frailty [3, 9, 11, 12]. According to the World Health Organization, older adults aged 65 years and older are recommended to engage in at least 150 min of moderate- or 75 min of vigorous-intensity aerobic physical activity and two days of muscle-strengthening activities a week [13]. Similarly, frail older adults are encouraged to perform aerobic and resistance training as such exercises address the hallmarks of frailty including muscle weakness, slowness, fatigue and low physical activity [14]. These types of exercises are often implemented at moderate- to vigorous-intensity levels in multicomponent exercise interventions, and several systematic reviews have shown their effectiveness among pre-frail and frail older adults [15–17]. However, given that fatigue and exercise intolerance are distinct features of frailty [1, 18], it may be challenging for some frail individuals to perform such recommended exercises. Hence, it is equally important to consider the safety, acceptability, and feasibility of exercise programs tailored to this specific population group.

Baduanjin (BDJ), also known as Eight-Section Brocades, is a low-intensity traditional Chinese Qigong exercise that involves eight simple movements in combination with meditative and breathing techniques [19, 20]. As a mind-body exercise, it is purported to regulate the body flow of ‘Qi’ (vital energy), which confers physical and cognitive function benefits [21–23]. Given the simplicity of its exercise routine, BDJ can be easily learned thus making it an appropriate exercise for populations with physical or cognitive impairment [23, 24]. Studies have found several health benefits of BDJ such as improved quality of life, physical performance, and fatigue levels in various clinical populations [25–27]. Albeit a small number of studies and poor methodological quality, a recent systematic review and meta-analysis reported the beneficial effects of BDJ in Chinese older adults aged 65 years and older with improvements in physical function, walking ability, and balance [28]. Hence, existing literature suggests that such an exercise intervention is potentially helpful for frail older adults.

We had previously demonstrated the feasibility and safety of implementing BDJ exercise program in the community for frail older adults through a single-group pilot study [20]. The study suggested possible improvements in physical and psychological measures, with knee extension strength, fear of falling and vital exhaustion showing the most significant changes. Therefore, BDJ has the potential to improve specific phenotypic components of frailty [1]. However, the lack of a control group limits the ability to determine the effects of BDJ among frail older adults. To date, there is a lack of randomized controlled trials to evaluate BDJ training in this specific older adult population group [24, 29]. Therefore, the aim of this study was to examine the effectiveness and implementation of a 16-week BDJ exercise intervention for community-dwelling older adults with varying frailty status in local senior activity centers. It was hypothesized that the exercise intervention would be effective to improve physical and psychological outcomes.

## Methods

### Study design

This study employed a two-arm, multicenter assessor-blinded parallel-group randomized controlled trial study design. The trial was conducted at three senior activity centers between February 2021 and May 2023 (ClinicalTrials.gov Identifier: NCT04549103). Participants enrolled at each center were randomly allocated to either the intervention group (IG) or control group (CG) with a 1:1 allocation ratio. Randomization sequence was generated based on a computerized block randomization with block sizes of 4 and was concealed from personnel involved in recruitment of participants. Ethical approval was obtained from the National Healthcare Group Domain Specific Review Board (2020/00100) and all participants provided written informed consent prior to study participation. This trial was reported in accordance with the Consolidated Standards of Reporting Trials guidelines [30].

### Participants

Study participants were recruited at the three senior activity centers through convenience sampling methods. Participants were considered eligible for the study if they (1) were aged 55 years and older; (2) had low muscle strength; (3) were able to ambulate independently with no other physical limitations affecting study participation and adherence; (4) were able to understand basic instructions; and (5) had generally sedentary lifestyles. Low muscle strength was defined as handgrip strength less than 28 kg and 18 kg in men and women, respectively according to the Asian Working Group for Sarcopenia 2019 consensus [31]. Sedentary lifestyle was defined as participating in sitting activities at least five days per week for more than four hours per day on average [32]. Participants were excluded if they met any of the following exclusion criteria: (1) participating in other intervention studies, (2) engaging in moderate or vigorous intensity exercise, (3) performing regular Tai Chi or Qigong exercises, (4) have severe audio-visual impairment, (5) diagnosed with cognitive impairment and/or history of neurological disorder, (6) diagnosed with postural hypotension, (7) unable to participate for the full duration of the study, (8) unable to come to the center with/without personal assistance, and (9) deemed not suitable to participate by a medical doctor. All participants underwent physical examination by a doctor for pre-exercise medical clearance before study enrollment.

### Intervention group

The IG underwent a 16-week BDJ exercise program that was developed and delivered by the local Qigong association [20]. The training program consists of 44 sessions of 60 min each over 16 weeks. Each session was conducted in an indoor group setting at each center by two certified instructors. In the first four weeks, the training sessions were conducted twice per week with the focus on familiarizing the participants with each of the BDJ routine’s eight movements, which have been previously detailed [20]. In the following 12 weeks, the sessions were conducted thrice per week in which the participants were instructed to practice the whole BDJ routine. Participants were expected to perform four sets of the BDJ routine during each session and were also taught meditative and breathing techniques. In addition, participants were given an instructional video and encouraged to practice the BDJ routine independently outside class to reinforce learning. To ensure safety, a chair was placed within arm’s reach of each participant if rest was needed. If preferred, participants performed the exercise in a seated position. Participants’ blood pressure, arterial blood oxygen saturation and heart rate were monitored by a research coordinator at the start and end of each training session. Participants did not proceed with the training session if either (1) abnormal blood pressure (systolic blood pressure ≥ 130 mmHg or diastolic blood pressure ≥ 80 mmHg), (2) low blood oxygen saturation < 95%, (3) high heart rate (≥ 90 beats per minute), (4) giddiness, or (5) any form of discomfort was present.

### Control group

Participants in the CG received a 60-minute health education talk once every four weeks over the 16-week period. The topics for the education talk included physical and mental function maintenance, relationship management, health risks and diseases management, and general well-being using traditional Chinese medicine. They were also instructed to maintain their usual physical activity levels. The CG had the opportunity to attend the same exercise program after completing the post-intervention assessment.

### Outcomes

All outcome measures were conducted at baseline and 4-month follow-up by trained assessors who were blinded to the participants’ group allocation.

#### Primary outcome measures

**Knee extension strength** Participants’ knee extension strength was measured in kilograms of maximal force exerted using a digital dynamometer gauge (Model 12–0342, Baseline Corporation, Irvington, NY). Participants were instructed to extend their legs against a spring gauge strapped 10 cm above the ankle joint while seated with the hip and knee joint angles positioned at 90 degrees. Two trials were administered for each leg and the highest of four readings were used for analysis.

**Maastricht Questionnaire (MQ)** MQ is a validated measure of vital exhaustion [33]. It consists of 21 items that measure dimensions of excessive fatigue, increased irritability, and feelings of demoralization. The overall vital exhaustion is computed by summing up the responses, which ranges from 0 to 42 and higher scores indicate greater vital exhaustion.

**Falls efficacy scale-international** The falls efficacy scale-international questionnaire is a validated 16-item questionnaire that measures the fear of falling among older adults during physical and social activities inside and outside the home [34]. Participants responded to a 4-point Likert scale ranging from 1 (not at all concerned) to 4 (very concerned). Scores range from 16 to 64 points with higher scores indicating greater fear of falling.

#### Secondary outcome measures

**Physiological profile assessment (PPA)** Participants’ physiological falls risk was measured using the PPA short version, which consists of five components [35]:

- Visual contrast sensitivity was assessed using the Melbourne Edge Test, where twenty circular patches with decreasing edge contrast were positioned about 40 cm from the participant. Participants selected from four options for each patch, and the lowest contrast sensitivity was based on the final correct response.
- Lower limb proprioception was assessed using a lower limb matching task. Participants, with eyes closed, matched their lower limbs on either side of a protractor-marked acrylic sheet. Five trials were administered with the average degree of deviation recorded.
- Knee extension strength was measured using the protocol mentioned above.
- Reaction time was measured using a hand reaction time test, where participants pressed a modified computer mouse switch in response to a light stimulus. Reaction time was measured in milliseconds using a built-in timer, and ten trials were administered with the average reading recorded.
- Postural sway was measured using a sway meter (Neuroscience Research Australia, New South Wales, Australia) that measures body displacement at waist level. Participants stood as still as possible with eyes open on a foam mat for 30 s. A 40-centimeter rod with a vertically mounted pen was attached to the participant’s lower back to record the postural sway on a sheet of graph paper, and the total sway area in square millimeters was recorded.

A composite score was computed based on weighted scoring of the five components using the NeuRA FallScreen Falls Risk Calculator (https://fallscreen.neura.edu.au/), and higher scores indicate greater risk of falls.

**Handgrip strength** Handgrip strength was measured with a hand dynamometer (Jamar Plus+, Patterson Medical, Cedarburg, WI). Participants were instructed to squeeze the dynamometer with maximum effort in a seated position with their arms at their sides and elbows flexed at 90 degrees. Two trials were administered for each arm and the highest of four readings was used for analysis.

**6-meter fast gait speed** Participants were instructed to walk six meters over a level surface with an additional one meter for acceleration and one meter for deceleration at a walking pace as fast as possible with or without walking aids. Two trials were administered, and the mean values were recorded for analysis.

**Timed up and go (TUG)** TUG is a reliable and valid assessment of mobility in older adults [36]. The test measures the time taken for participants to rise from a seated position, walk three meters at a comfortable speed, make a turn, walk back, and return to a seated position. Two trials were administered, and the mean values were recorded for analysis.

**30-second sit-to-stand** This test is a measure of functional physical performance that is influenced by both physiological and psychological processes [37]. Participants were instructed to perform repeated chair stands using a chair without arms. With their arms folded across their chest, number of completed full stands without using arms within a 30-second period was recorded for analysis.

**EQ-5D-5 L** Health-related quality of life was measured using the EQ-5D-5 L index score [38]. The score ranges from − 0.59 to 1 and is computed based on five dimensions (mobility, self-care, usual activities, pain/discomfort, and anxiety/depression) with higher scores indicating better quality of life.

**Geriatric depression scale** The geriatric depression scale is a validated 30-item questionnaire that measures depression in older adults [39]. Participants responded to each item by answering yes or no. Scores range from 0 to 30 with higher scores indicating greater severity of depression.

**Frailty status** Frailty was determined using the Fried’s phenotype criteria, which characterizes frailty based on five components: weakness, unintentional weight loss, slowness, exhaustion and low physical activity [1]. Weakness was determined using the Asian Working Group for Sarcopenia’s criteria of handgrip strength less than 28 kg and 18 kg in men and women, respectively [31]. Unintentional weight loss was defined by either body mass index less than 18.5 kg/m^2^ or self-reported weight loss of at least 4.5 kg in the past six months. Slowness was identified using the 6-meter fast gait speed with specified cut-offs based on gender and height: 0.65 m/s for men ≤ 173 cm and women ≤ 159 cm, and 0.76 m/s for men > 173 cm and women > 159 cm. Exhaustion was self-reported through a 3-item questionnaire adapted from the SF-12 questionnaire [40]. Low physical activity was assessed using the Longitudinal Ageing Study of Amsterdam Physical Activity Questionnaire [41]. Low physical activity was defined as energy expenditure less than 383 kcal per week and 270 kcal per week for men and women, respectively. Presence of each of the five components was assigned one point, and the categorization of frailty status was defined as robust (0 point), pre-frail (1–2 points), and frail (3–5 points) [1].

### Evaluation of program implementation

A participant feedback questionnaire was administered to all IG participants who completed the intervention at 4-month follow-up. Participants were asked to rate their experience with the BDJ intervention by indicating the degree of agreement with the questionnaire items on a 4-point Likert scale ranging from 1 (strongly disagree) to 4 (strongly agree). In addition, participants responded to open-ended questions on the motivating factors, perceived benefits and challenges and recommendations for the exercise program.

### Sample size calculation

The present study’s sample size was calculated based on the estimated effect from our previous pilot study [20]. Based on a priori power analysis (G*Power 3.1.9.3) using a statistical power of 0.90 and error probability of 0.05, a sample size of 54 participants was required to detect an effect size of *d* = 0.6 in knee extension strength between CG and IG. Assuming a 10% dropout rate, a sample size of 60 participants was targeted.

### Statistical analysis

Analyses were conducted based on intention-to-treat principle, and all participants with completed baseline outcome measures were included in the analyses. Independent sample *t* tests and chi-square tests were performed to examine differences in baseline measures between CG and IG for continuous and categorical variables, respectively. Linear mixed-effect models were employed to examine the changes in outcome measures between baseline and 4-month follow-up across the two groups. The models included group, time, and group x time interaction as fixed effects, and random intercepts were included for each participant to account for within-subject correlations. All mixed-effect models were adjusted for age, gender, education, living status, and number of comorbidities. Post-hoc pairwise comparisons were conducted to examine the main effect of time in respective groups. Statistical significance level was set at *p* < .05 and all analyses were performed using R statistical software, version 4.1.2 (R Foundation for statistical computing, Vienna, Austria).

## Results

### Participant characteristics

**Fig. 1:**
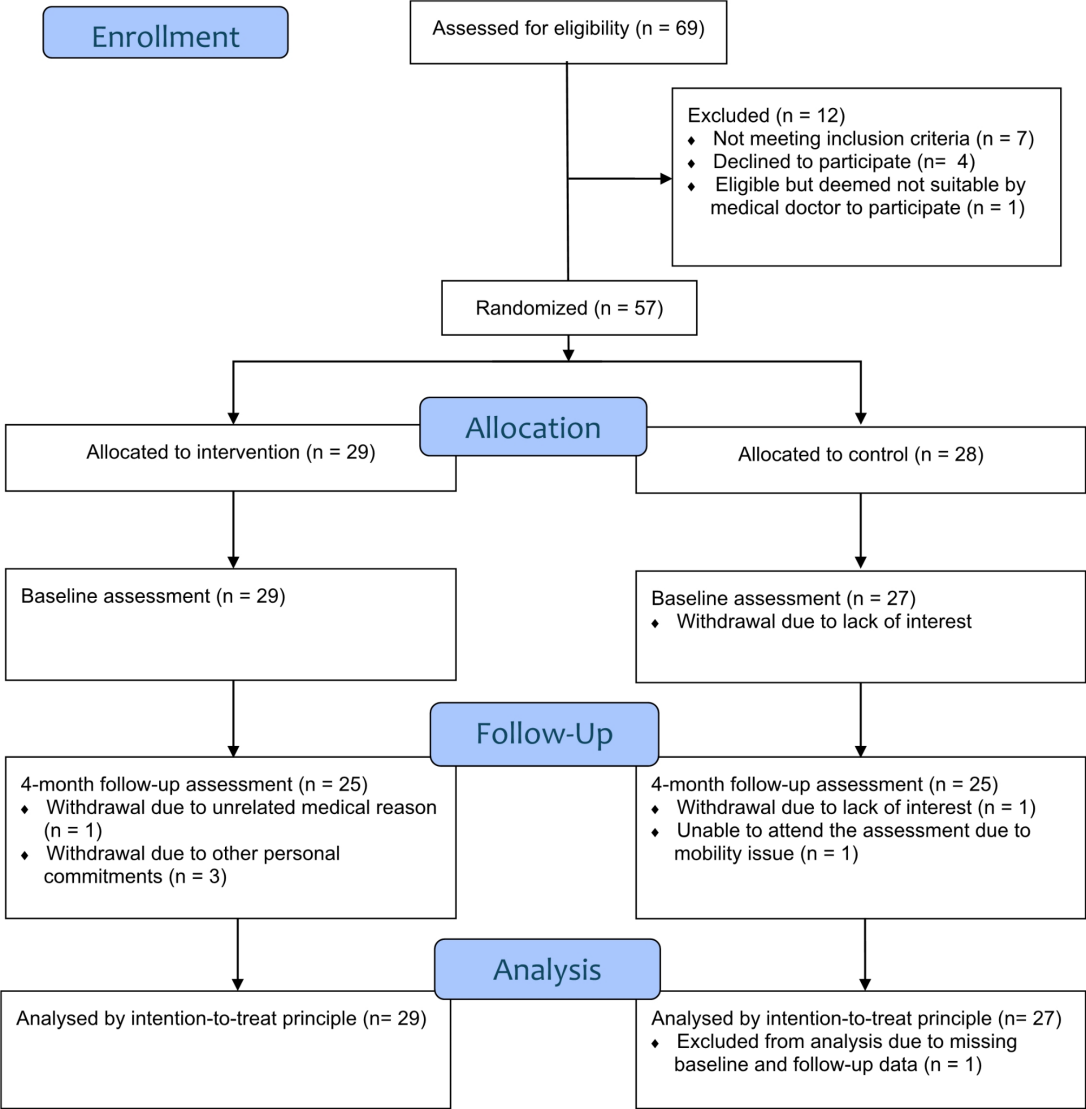
Study flow diagram

Four batches of older adults were recruited through Tsao Foundation with community partners from three senior activity centers. Amongst the 69 older adults referred to the study, seven did not meet the inclusion criteria, one was deemed unsuitable by a medical doctor, and four declined to participate. 57 participants enrolled in the study and were randomized into either the CG (*n* = 28) or IG (*n* = 29). Seven participants dropped out from the study due to lack of interest (*n* = 2), conflict in schedule with other personal commitments (*n* = 3), and unrelated medical conditions (*n* = 2). A total of 56 participants with available baseline data were included in the final analysis sample. Figure 1 showed the participant flow.

Table 1 showed the baseline demographic characteristics of both CG and IG. The participants were mostly women aged 72.8 (6.9) years. There were no statistically significant differences in demographic characteristics or outcome measures between IG and CG.

**Table 1:** Baseline characteristics of participants in both CG and IG

	Control Group (*n* = 27)	Intervention Group (*n* = 29)	*p*
**Age (years)**	72.6 (5.7)	72.9 (8.0)	0.856
**Gender** (***n***(%))			0.479
Female	25 (92.6%)	24 (82.8%)	
**Housing** (***n***(%))			1.00
< 3 rooms apartment	4 (14.8%)	4 (13.8%)	
**Living status** (***n***(%))			0.280
Alone	9 (33.3%)	5 (17.2%)	
**Education** (***n***(%))			1.00
< Primary education	12 (44.4%)	13 (44.8%)	
**Smoking history** (***n*** (%))			0.073
Current smoker	0 (0.0%)	0 (0.0%)	
Ex-smoker	0 (0.0%)	5 (17.2%)	
Non-smoker	27 (100.0%)	24 (82.8%)	
**Falls history**			1.00
Yes	14 (51.9%)	14 (48.3%)	
**Body mass index (kg/m** ^**2**^ **)**	25.1 (4.0)	23.6 (3.4)	0.127
**Comorbidities** (***n*** (%))			
Arthritis	10 (37.0%)	6 (20.7%)	0.291
Cardiovascular disease	1 (3.7%)	3 (10.3%)	0.656
Diabetes	4 (14.8%)	8 (27.6%)	0.402
Hyperlipidaemia	14 (51.9%)	18 (62.1%)	0.616
Hypertension	14 (51.9%)	17 (58.6%)	0.810
Osteoporosis	5 (18.5%)	6 (20.7%)	1.00
**Frailty status** (***n*** (%))			0.689
Robust	6 (22.2%)	4 (13.8%)	
Pre-frail	19 (70.4%)	22 (75.9%)	
Frail	2 (7.4%)	3 (10.3%)	

Data presented in n (%) or mean (SD)

### Outcome measures

Table 2 showed the outcome measures at baseline and 4-month follow-up for both IG and CG. Among the primary outcomes, there were no statistically significant interaction between group and time for knee extension strength (B = 1.63, 95% confidence interval (CI) [-0.73, 3.96], *p* = .180), MQ scores (B = -2.20, 95% CI [-5.64, 1.37], *p* = .224), and falls efficacy (B = 2.85, 95% CI [-2.22, 7.99], *p* = .279). Similarly, there were no statistically significant between-group differences in all secondary outcome measures.

**Table 2:** Outcome measures at baseline and 4-month across CG and IG

	Control (*n* = 27)			Intervention (*n* = 29)			Group x Time interaction		
	Baseline	4-month	*p*-value	Baseline	4-month	*p*-value	B (95% CI)	S.E.	*p*- value
**Primary Outcomes**									
KES (kg)	14.00 (4.36)	13.52 (4.29)	0.439	15.13 (5.98)	16.58 (6.25)	0.189	1.63 (-0.73, 3.96)	1.20	0.180
MQ	13.07 (8.15)	10.28 (7.36)	0.128	17.45 (7.89)	13.46 (10.05)	0.005*	-2.20 (-5.64, 1.37)	1.79	0.224
Falls efficacy	25.52 (10.39)	28.16 (7.69)	0.125	25.38 (10.80)	30.12 (11.74)	0.004*	2.85 (-2.22, 7.99)	2.60	0.279
**Secondary Outcomes**									
PPA	1.48 (1.46)	1.35 (1.40)	0.720	1.59 (1.96)	1.16 (1.81)	0.096	-0.34 (-0.99, 0.32)	0.33	0.316
HGS (kg)	18.29 (4.37)	17.59 (4.83)	0.496	18.65 (7.46)	20.32 (8.58)	0.127	1.38 (-0.25, 3.03)	0.84	0.105
6-m fast GS (m/s)	1.34 (0.38)	1.36 (0.30)	0.680	1.40 (0.39)	1.43 (0.34)	0.879	0.01 (-0.09, 0.11)	0.05	0.875
TUG (s)	11.71 (5.88)	10.58 (2.59)	0.588	12.37 (6.72)	10.94 (3.28)	0.217	-0.88 (-2.71, 1.01)	0.94	0.357
30-s sit-to-stand	14.41 (4.32)	15.60 (5.80)	0.389	13.75 (3.73)	14.75 (4.47)	0.496	-0.18 (-2.14, 1.76)	0.99	0.859
EQ-5D index score	0.83 (0.09)	0.85 (0.05)	0.265	0.81 (0.12)	0.80 (0.11)	0.955	-0.03 (-0.08, 0.03)	0.03	0.348
GDS	3.33 (2.54)	2.96 (2.35)	0.829	4.07 (3.53)	4.00 (3.39)	1.00	0.22 (-1.30, 1.77)	0.78	0.781
Frailty	1.19 (0.88)	1.00 (1.00)	0.723	1.52 (0.87)	1.24 (1.05)	0.228	-0.12 (-0.69, 0.46)	0.29	0.684

Model adjusted for age, gender, education, living status and comorbidities

Data presented in n (%) or mean (SD)

*B* unstandardized beta coefficient, *HGS* handgrip strength, *GDS* Geriatric depression scale, *GS* Gait speed, *KES* Knee extension strength, *MQ* Maastricht questionnaire, *PPA* Physiological profile assessment, *S.E.* Standard error, TUG Timed up and go test, *95% CI* 95% confidence interval, *p < .05

Post hoc pairwise comparisons revealed statistically significant within-group differences between baseline and 4-month follow up in IG. IG showed increased MQ scores by 4.31 points (95% CI [1.41, 7.20], *t*(25) = 3.06, *d* = 0.60, *p* = .005) but their fall efficacy scores also increased by 5.64 points (95% CI [1.97, 9.31], *t*(24) = 3.17, *d* = 0.63, *p* = .004). In contrast, no statistically significant differences were found in all outcome measures for CG.

### Compliance analysis

We performed additional analyses on a subgroup of participants with at least 75% attendance rate to assess the effects for individuals with good compliance to the intervention. A statistically significant interaction between group and time was found for MQ scores (B = -3.65, 95% confidence interval (CI) [-7.13, -0.16], *p* = .047).

### Implementation outcomes

Four different batches of older adults participated in the BDJ exercise program. There were no falls or major adverse events during the intervention for all batches of participants. The average participant attendance rate was 81.3%. Among the 25 IG participants who completed the exercise program, 19 (76.0%) attended at least 75% of the sessions and two (8.0%) participants achieved 100% attendance rate. Among the 44 BDJ exercise training sessions conducted for each batch of participants, the average class attendance was 77.6%. The median class attendance was 75.0%, and the interquartile range was between 71.4 and 87.5%. Reasons for absence were medical appointments, feeling unwell, and conflict in class schedule with other personal commitments.

The curriculum of the 16-week BDJ exercise program was delivered as planned for all batches of participants. However, due to social distancing advisory during COVID-19 pandemic, the intervention was partly delivered in hybrid modes for one cohort of participants. Two participants joined the exercise training via a live video-conferencing platform. They were able to follow the program and no adverse events occurred during the sessions.

The participant feedback survey was administered to 25 IG participants who completed the study. All participants agreed that they had positive experiences with the BDJ exercise program with perceived physical, psychological, and social benefits. They also indicated that they could follow the program and independently perform the BDJ exercise routine (Table 3). The common reported motivations to join the BDJ exercise program were opportunities to exercise and socially interact with others. Several participants felt more energetic through joining the program, crediting their improved energy levels to the breathing exercises they practiced. Nevertheless, some participants faced challenges during the 16-week program, which included difficulty in following the routine during initial stages and executing certain movements due to joint pain. When asked about how the exercise intervention could be improved, the majority suggested more exercise movements to add variation to the program.

**Table 3:** Responses on intervention participant experience (*n* = 25)

Questionnaire Items	Score
I enjoyed the program.	3.60 (0.50)
The instructor conducted the program in an engaging manner.	3.60 (0.50)
The instructor is knowledgeable and able answers my questions.	3.64 (0.49)
I am able to follow the exercises.	3.68 (0.48)
The intensity of the exercise was manageable	3.64 (0.49)
The instructor gives me to ability to perform the exercises on my own.	3.52 (0.51)
The program was relevant and useful to my activities of daily living.	3.48 (0.51)
After starting Baduanjin program, I feel stronger and more confident in my daily living.	3.32 (0.48)
After starting Baduanjin program, I felt more energetic and able to do more things in the day.	3.32 (0.56)
After starting Baduanjin program, my social interactions with others improved.	3.20 (0.50)
After starting Baduanjin program, I feel happier and more joyful.	3.44 (0.51)
After starting Baduanjin program, I am motivated to continue practising the exercise in the future.	3.56 (0.51)
After starting Baduanjin program, I will recommend the Baduanjin program to others.	3.60 (0.50)

Data presented in mean (SD)

## Discussion

The present study examined the effectiveness and implementation of a 16-week BDJ exercise intervention for community-dwelling older adults with varying frailty status. BDJ was found to be a safe, feasible, and acceptable exercise intervention that can be successfully implemented in community settings. Contrary to our hypothesis, the lack of statistically significant differences in all outcome measures between IG and CG suggests that the effectiveness of BDJ in predominantly pre-frail and frail older adults is inconclusive. Exploratory compliance analyses revealed that BDJ could potentially alleviate vital exhaustion among participants with good adherence to the exercise intervention.

BDJ has been posited as a low-intensity exercise with several potential health benefits in different population groups [21–23] including older adults [28]. In a previous single-arm feasibility study, we reported improvements in physical and psychological outcomes among a small group of frail older adults after the same 16-week BDJ exercise intervention [20]. In alignment with these previous studies, the present study found that the direction of average effect estimates favors the BDJ intervention for most outcome measures. However, the 95% CI indicates that these estimates lack precision. Thus, we could not rule out the null hypotheses that there is no difference between IG and CG.

Counter to expectation, the present study findings suggest that the magnitude of BDJ’s effects on physical, psychological and frailty outcomes in community-dwelling older adults are likely modest. Comparison of change scores between both groups revealed that the effect sizes for all outcome measures are less than *d* = 0.4, or small to moderate [42]. Particularly, estimated effects on gait speed (*d* = 0.05), sit-to-stand performance (*d* = 0.03), and depression scores (*d* = 0.04) are likely too small to have clinical relevance. Such magnitudes are smaller than the synthesized effects found in a recent meta-analysis study that examined the effects of BDJ in Chinese older adults population [28]. As the meta-analysis included studies conducted in institutionalized settings and all outcome assessments were not blinded, the contrasting findings could likely be attributed to differences in study population and methodological quality. Our trained assessors were blinded to group allocation in the present study. Therefore, our findings contribute to existing literature by providing robust evidence regarding the effects of BDJ in community-dwelling older adults who are predominantly prefrail or frail.

Preserving and improving muscle strength are imperative among older adults, especially for those who are frail. Muscle weakness, which is a hallmark and recognized to be the first manifestation of frailty [43], is associated with greater risk of falls [44]. BDJ is purported to have beneficial effects on muscular strength as it encompasses both upper and lower body isometric exercises while maintaining the postures in its routine [22]. However, we did not find any statistically significant changes in muscular strength and physiological falls risk. As most participants are pre-frail and a few are robust, it is possible that the intensity of BDJ is not sufficiently high to elicit observable improvements in our study sample. The lack of effects on physical performance among community-dwelling older adults has also been previously reported in similar low-intensity interventions such as Tai Chi [45–47]. While some advocate for low-intensity physical activities for older adults to improve adherence [48], it is equally important to ensure that the exercise intensity is adequate to maintain and improve physical function. Given that poor muscle strength and frailty are both associated with falls [44, 49], higher intensity resistance training may be required to evoke noticeable improvements in frail older adults [11].

Exercise interventions have been demonstrated to have a small to moderate reduction in fear of falling among community-dwelling older adults [50]. Surprisingly, the IG was found to report increased fall efficacy scores after the 16-week BDJ intervention, indicating greater fear of falling. This surprising result could plausibly be attributed to the profile of study participants, in which almost half (48.3%) of IG had a history of falls. Given that history of falls is associated with greater fear of falling [51], the IG might have developed heightened anxiety of falling while engaging in the BDJ intervention. It has been shown that long-term exercise participation itself may not necessarily reduce fear of falling in older adults [52]. Thus, it might be necessary to supplement exercise programs with additional interventions to diminish the fear of falling in older adults.

Based on traditional Chinese medicine theory, Qigong is postulated to integrate the mind, body, and spirit through regulation of Qi to improve physical and mental well-being [53]. Such regulation of vital energy in Qigong has been found to relieve fatigue symptoms in various patient populations [54]. BDJ is designed to facilitate integrated Qi movements through its eight simple movements routine [19]. Notably, in corroboration with findings from our previous feasibility study [20], we observed a moderate to large improvement in exhaustion scores in the IG in the present study. In addition, subgroup analysis of participants with at least 75% attendance rate yielded a statistically significant interaction between group and time for MQ scores. This suggests that with good adherence, the 16-week BDJ intervention was effective in reducing vital exhaustion. Considering that most transitions to frailty involved manifestation of exhaustion symptoms [43], BDJ could be potentially useful to manage this aspect of frailty.

Translational research is necessary to bridge the gap between research and practice [55]. Frailty interventions need to be implemented and evaluated in real-world settings to for effective translation [56]. We showed that BDJ exercise program can be successfully implemented in local senior activity centers. Although there were some dropouts (13.7%), the exercise intervention exhibited good adherence with 81.3% average attendance. Social interaction can improve participation in community-based programs [57]. Indeed, many IG participants cited social interaction opportunities as their motivation and appeal of the BDJ program, which was conducted in group settings. Even though some participants opted for some live stream sessions during COVID-19, they had opportunities for social interaction when participating in person and also had some limited interaction during live stream. The simple BDJ routine makes it a suitable exercise for populations with physical or cognitive challenges [23, 24]. It can be practiced seated by persons using wheelchairs. The participant feedback survey revealed that all IG participants had positive experiences with the exercise intervention. While some cited initial challenges in learning the BDJ routine, all participants including one wheelchair user reported that they were confident of independently performing the BDJ exercise routine at the end of the 16-week intervention. Importantly, the absence of major adverse events suggests that BDJ is a safe exercise intervention for frail older adults when implemented under supervision of a qualified instructor. However, caution must be taken to manage participants with joint pains.

There are a few limitations to the present study. First, the study sample included some robust older adults. While low muscle strength was employed as the inclusion criteria to attempt to recruit a homogenous group of pre-frail and frail older adults, one male and twenty female participants exhibited unexpectedly higher handgrip strength during the baseline assessment, with ten of the females classified as robust. Although the sample is representative of the community-dwelling older adult population, this deviation may limit the generalizability of the present study’s findings to the target population of frail older adults. Second, this study consists of community-dwelling older adults. Thus, the findings may not generalize to frail older adults in institutionalized settings. In addition, while in concordance with similar previous studies conducted in the community [58, 59], readers should exercise caution when generalizing the results, as the sample was largely female. Considering that there are sex differences in adaptive responses to exercise [60], further studies with a more balanced gender distribution are needed to explore potential differences in the physiological mechanisms underlying the effects of exercise. Third, the scope of this study was limited to examining the short-term effects of the BDJ intervention. Given that low-intensity exercises encourage exercise adherence [61] and thus plausibly confer health benefits in a longer time horizon, future studies could explore the effects of BDJ over an extended period. Last, this study only examined the effectiveness of BDJ on physical and psychological outcome measures. As a mind-body exercise, previous studies have demonstrated the potential beneficial effects of BDJ on cognitive measures [23, 24]. Thus, the effects of BDJ on cognitive function among frail older adults warrant further attention.

## Conclusion

This study demonstrated that BDJ is a safe, feasible, and acceptable intervention that can be successfully implemented in neighborhood senior activity centers for older adults with varying frailty status. With good adherence to the intervention, BDJ is potentially effective in reducing exhaustion. However, the present study’s results suggest that the effectiveness of BDJ on physical performance, psychological measures, and frailty in community-dwelling older adults is equivocal. Exercise interventions with higher intensity levels are needed to elicit effects of greater magnitude.

## Data Availability

The datasets used and/or analyzed during the current study are available from the corresponding author on reasonable request.

## Abbreviations

BDJ: Baduanjin
CG: Control group
IG: Intervention group
MQ: Maastricht questionnaire
PPA: Physiological profile assessment
TUG: Timed up and go test
